# Supplementary material for: Hydrogen sulfide stimulates Mycobacterium tuberculosis respiration, growth and pathogenesis
Source: Nat Commun. 2020 Jan 28;11:557. doi: 10.1038/s41467-019-14132-y (PMC6987094; doi:10.1038/s41467-019-14132-y)
Supplement: Supplementary file 1 — Supplementary Information [file 41467_2019_14132_MOESM1_ESM.pdf]

Supplementary Information

Hydrogen sulfide stimulates *Mycobacterium tuberculosis* respiration, growth and pathogenesis

Saini, *et al*.

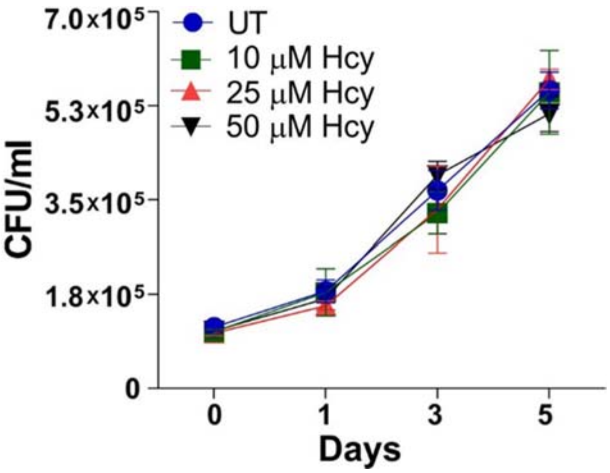

Supplementary Figure 1. Effect of homocysteine (Hcy) on *Mtb* infection. Peritoneal macrophages treated with Hcy have similar bacterial recovery as that of untreated (UT) cells. Data are representative of assays performed at least twice. Data are shown as mean ± SEM and were analyzed using one-way ANOVA with Sidak's multiple comparisons test to determine differences compared to UT at each time point. No statistically significant differences were observed between treatment groups at any time point (n=4 per group).

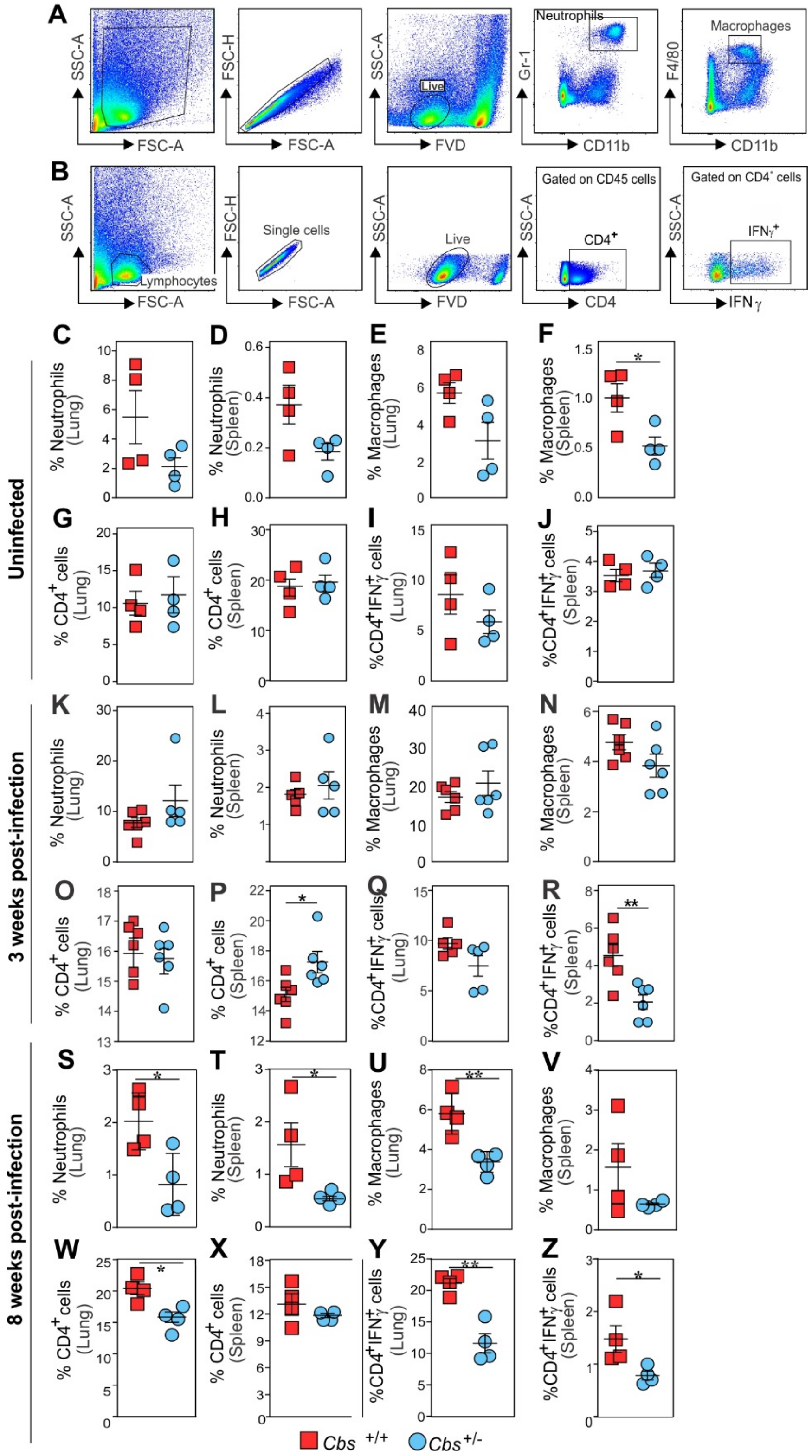

Supplementary Figure 2. Gating strategy and immune responses in WT and *Cbs*$^{+/-}$ mice. (**A, B**) The sequential flow cytometry gating strategy used for the identification of murine myeloid and lymphoid cell populations. Percentage distribution of macrophages (CD11b^{+}F4/80^{+}), neutrophils (CD11b^{+}Gr-1^{+}), CD4^{+} T-cells and CD4^{+}IFNγ^{+} T-cells in the lungs and spleens of uninfected **(C-J)** and 3 weeks **(K-R)** and 8 weeks **(S-Z)** post *Mtb*-infected WT and *Cbs*$^{+/-}$ mice. Data areshown as mean ± SEM (n=4-6 animals per group). Data were analyzed using unpaired parametric t-test. **P* < 0.05, ***P* < 0.

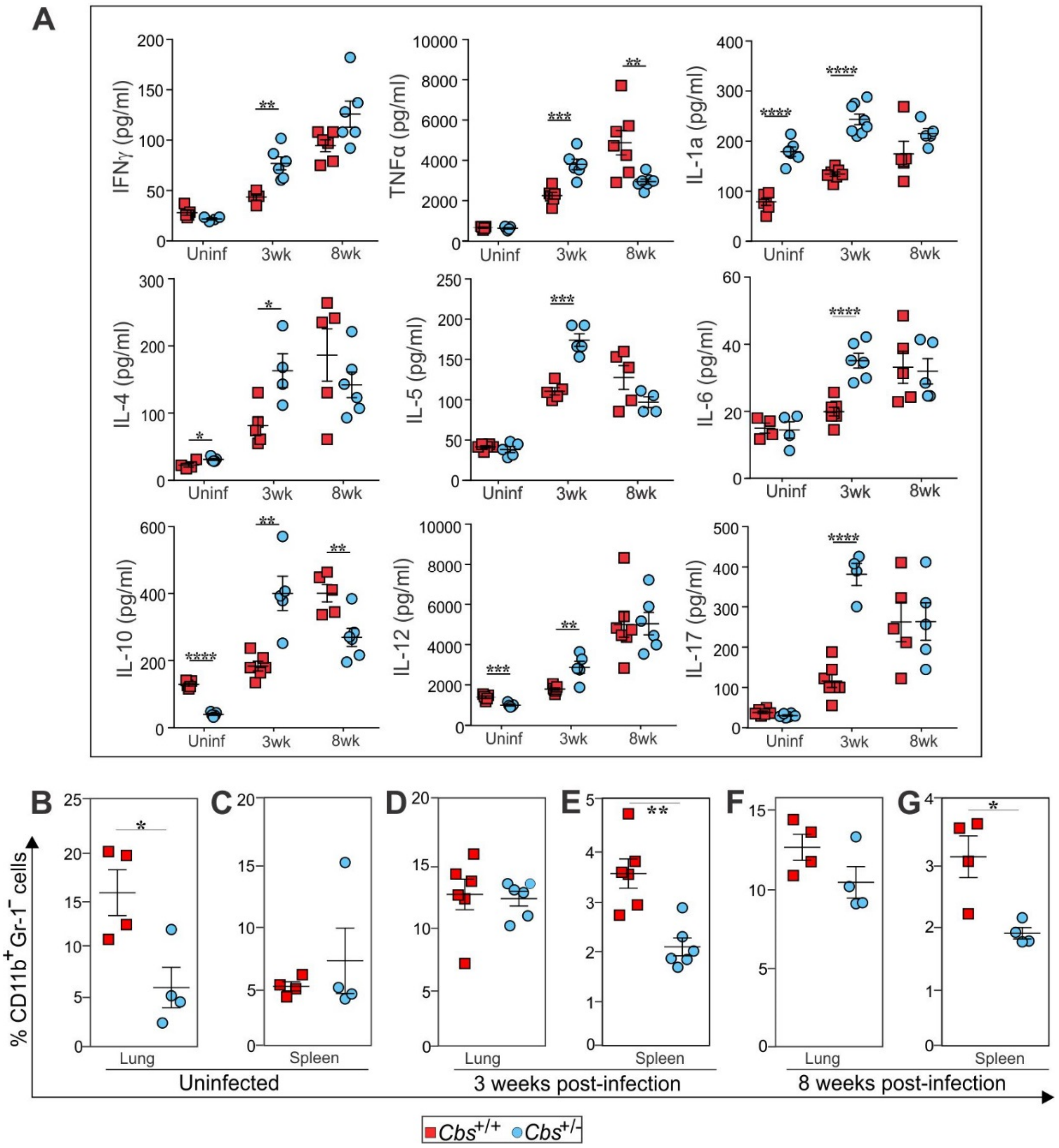

Supplementary Figure 3. Cytokine analysis and flow cytometric characterization of CD11b$^+$Gr-1$^-$ Cells in *Mtb*-infected mice. (**A**) Cytokine levels in the sera of mice without or with infection (3 wk and 8 wk). For the analysis of immune cells, the sequential gating strategy represented in Supplementary Figure Fig. 2A, B was used. Of the total number of live CD11b+ cells, neutrophils (CD11b$^+$Gr-1$^+$) were gated out and the remaining CD11b$^+$ cells were plotted. Percent CD11b$^+$Gr-1$^-$ cells in the lungs and spleens of (**B**, **C**) uninfected and *Mtb*-infected *Cbs*$^{+/+}$ and *Cbs*$^{+/-}$ mice at (**D**, **E**) 3 weeks and (**F, G**) 8 weeks post infection. Data are shown as mean ± SEM (4-7 animals per group) and were analyzed by unpaired parametric t-test to determine differences between genotypes in each organ or time point. **P* < 0.05, ***P* < 0.01, ****P* < 0.001, *****P* < 0.0001

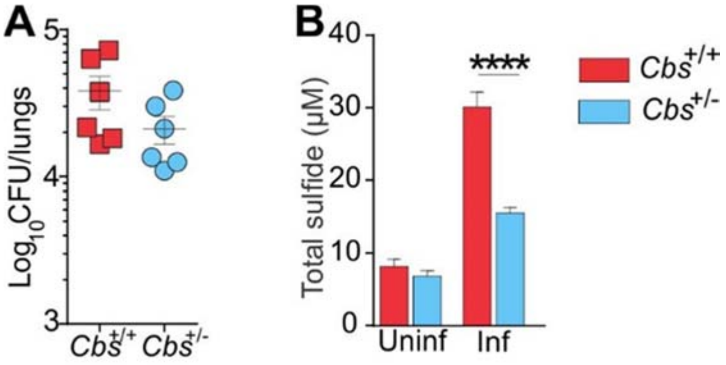

Supplementary Figure 4. CFU recovery and serum sulfide 3 weeks post-infection. (**A**) CFU recovery from *Mtb*-infected WT and *Cbs*$^{+/-}$ mice lungs at 3 wk post-infection. Data represent the mean ± SEM (n=6 animals per group). (**B**) Total sulfide levels in the sera of uninfected and infected mice at 3 wk post-infection. Data represent the mean ± SD (n=6 animals per group). All data were analyzed by unpaired parametric t-test to detect differences between genotypes under each condition.

****$P < 0.0001$

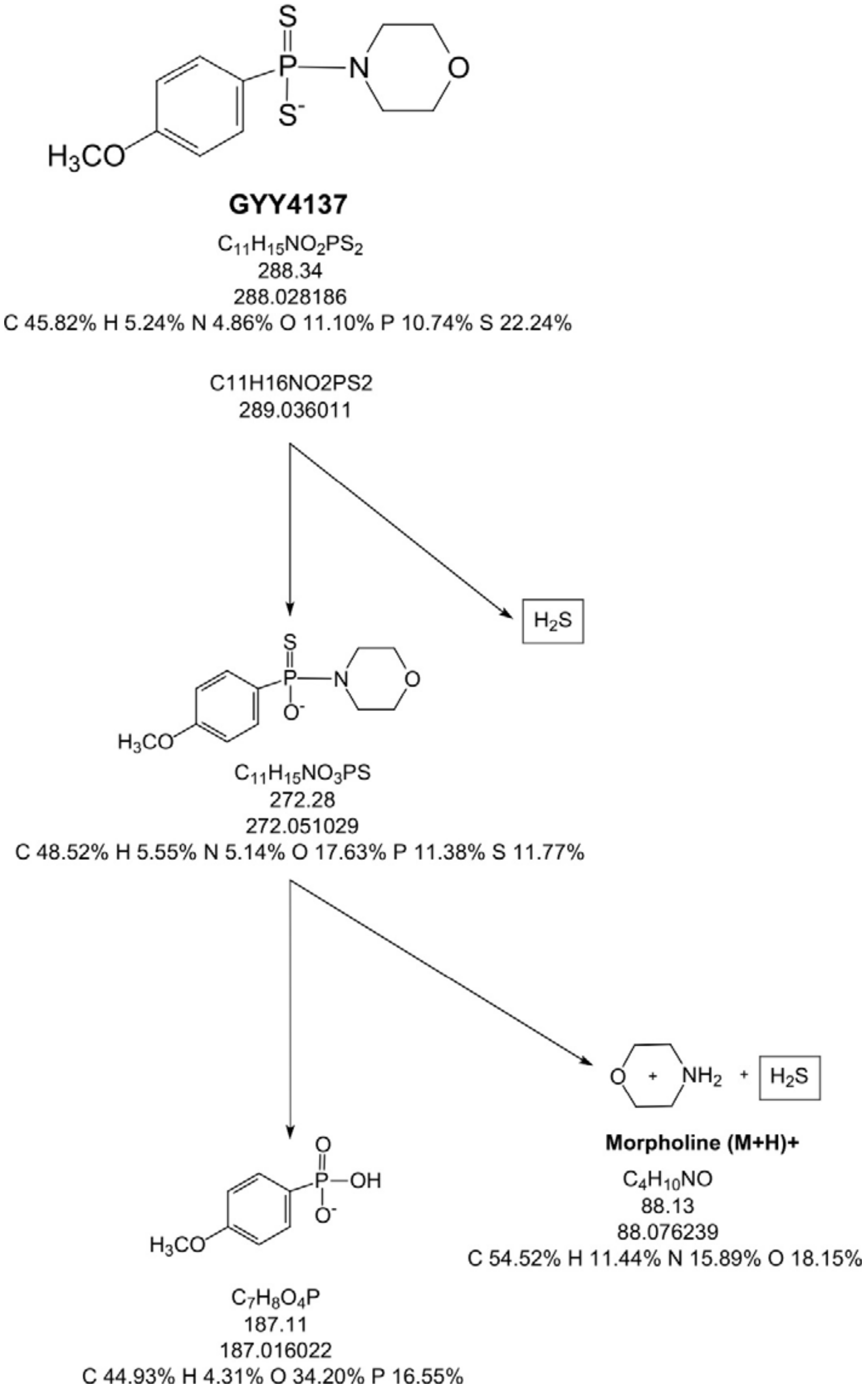

Supplementary Figure 5. Decomposition of GYY4137 into its corresponding breakdown products. High-resolution mass spectrometry was used to identify the breakdown products of GYY4137, with *m/z* of 272.05 $(M-H)^-$, 187.02 $(M-H)^-$ and 88.08 (morpholine $M+H)^+$ from *Mtb* cells exposed to GYY4137. Although the product ion 272.05, thought to be relatively unstable, was not found, both 187.02 and 88.08 ions were identified.

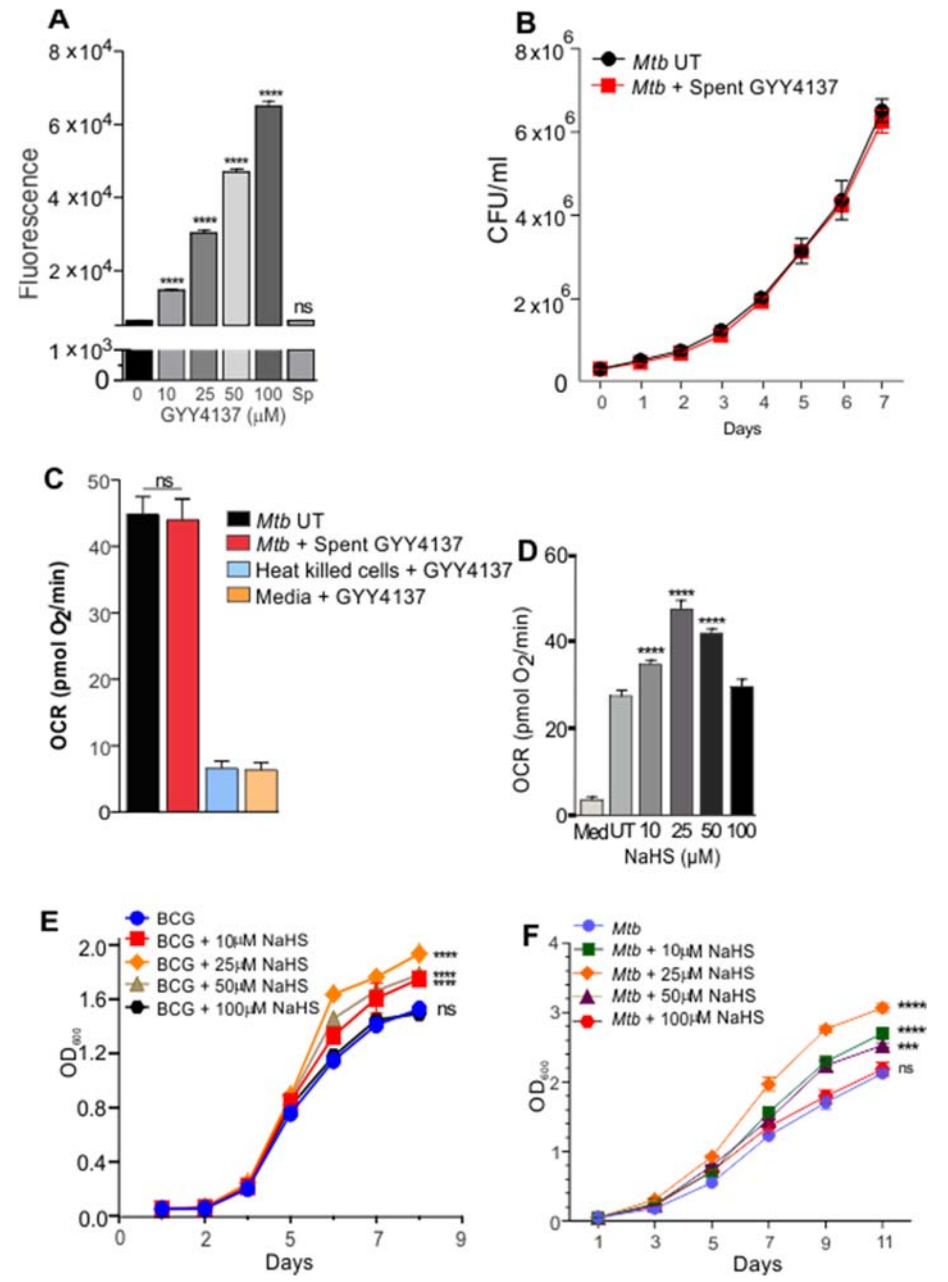

Supplementary Figure 6. Growth and bioenergetic profiles of mycobacterial cells receiving spent donor GYY4137 or NaHS. (A) Ratiometric quantitation of GYY4137-generated H_2S using the H_2S-specific fluorescent probe WSP1 in mycobacterial culture medium (n=8 per group). Spent (Sp) GYY4137 does not generate H_2S. (**B**) CFU-based growth profiles of *Mtb* cells performed in duplicate. Untreated (UT) *Mtb* cells received vehicle only while exposed cells received spent GYY4137 (25 μM). (**C**) OCR of live and heat-killed *Mtb* cells. Heat-killed cells and wells with media only (Med) received active GYY4137 (n=6-10 per group). (**D**) OCR of *M. bovis* BCG cells exposed to various concentrations of NaHS in an Oroboros O2-K chamber (n=4-6 per group). Untreated (UT) cells received vehicle without NaHS. Media only (Med) controls received 25 μM NaHS without cells. (**E**) Growth profiles of *M. bovis* BCG and (**F**) *Mtb* cells exposed to different concentrations of NaHS (n=3 per group). Data represent the mean ± SEM (**A**) and (**F**) or mean ± SD (**B**)-(**E**) and were analyzed using One-way ANOVA with Sidak's multiple comparisons test (**A, C, D**), Holm-Sidak multiple comparisons test (**B**), or two-way ANOVA with Tukey's multiple comparisons test (**E**) and (**F**).

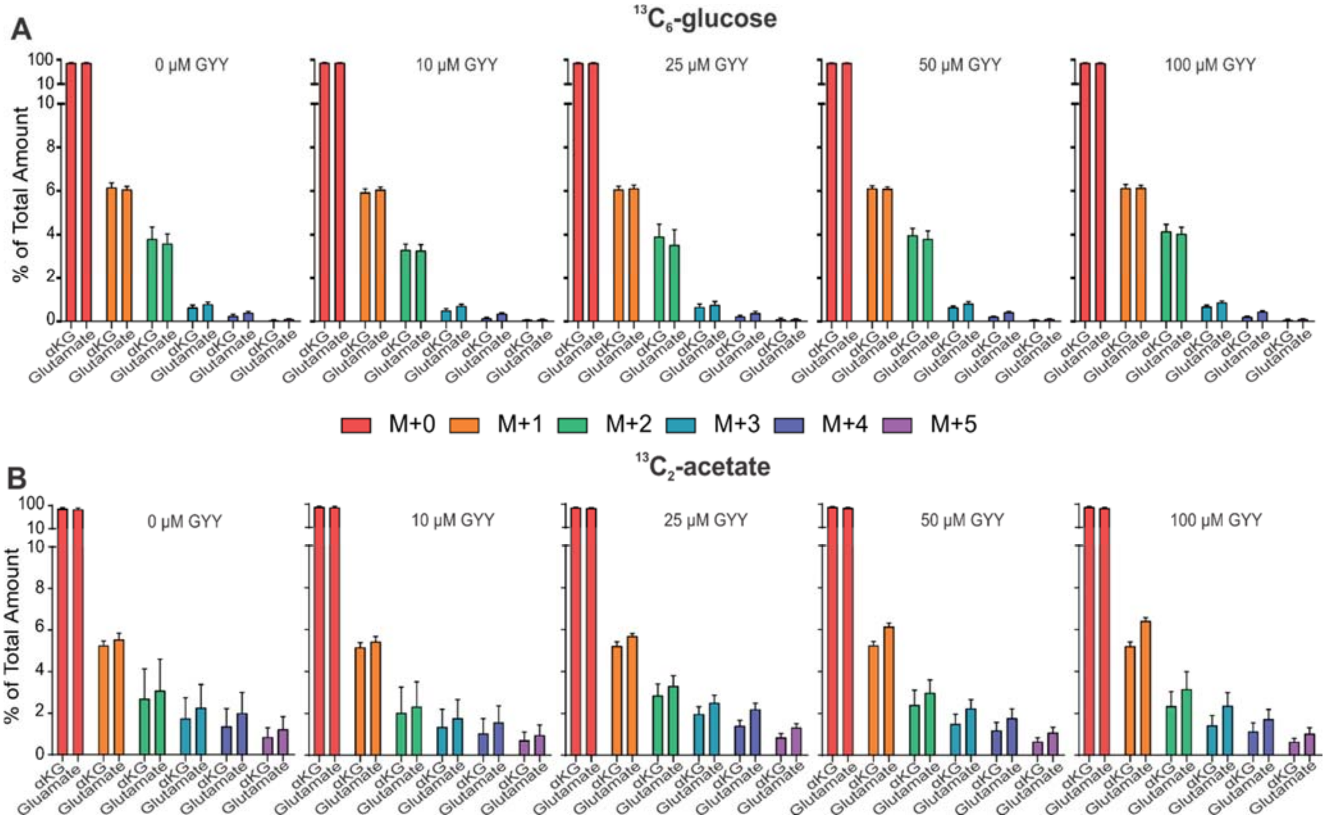

Supplementary Figure 7. Metabolic steady state during GYY4137 exposure. Isotopologue distribution of α-ketoglutarate (αKG) compared to the isotopologue distribution of glutamate at all GYY4137 concentrations used in the presence of $^{13}C_6$-gluose (**A**) and $^{13}C_2$-acetate (**B**) as carbon sources. There is no significant difference between the isotopologues of α-ketoglutarate and glutamate showing that the TCA cycle remains in steady state over the range of GYY4137 concentrations used. Data sets are shown as the mean ± SD (n=4 per group) and were examined using two-way ANOVA with Sidak's multiple comparisons test.

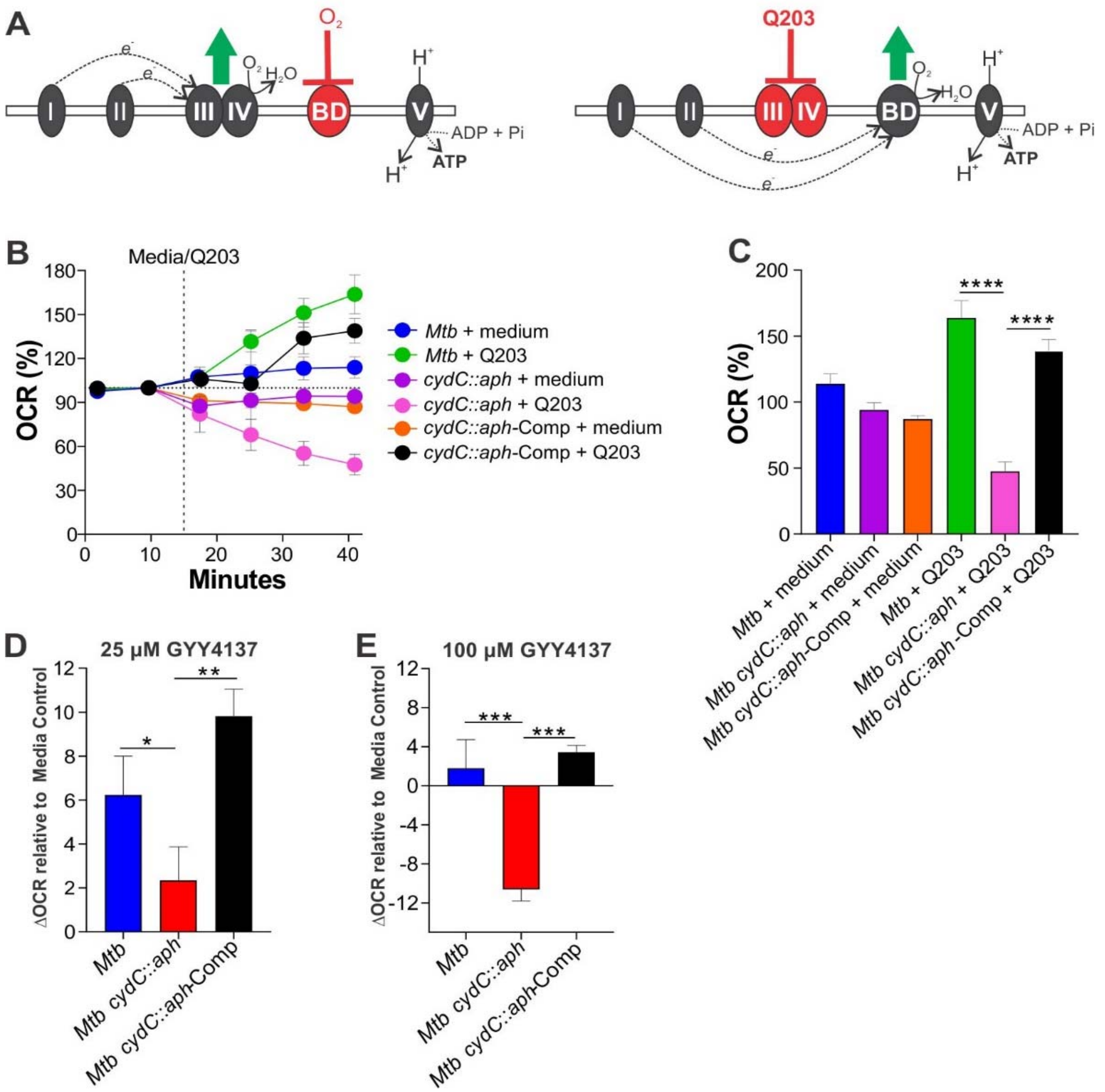

Supplementary Figure 8. Genetic complementation of *Mtb cydC::aph*. (**A**) CytBD is functional under hypoxic conditions, whereas cytochrome *bc₁/aa3* (Complex III/IV) is functional under normoxic conditions. Complementation of *Mtb cydC::aph* was demonstrated using Q203, which inhibits cytochrome bc_1 under normoxic conditions to reroute electrons to CytBD and is evident by an increase in respiration that can be measured by the Agilent Seahorse XF96 Extracellular Flux Analyzer (**B**) and (**C**). Exposure of WT *Mtb* cells to Q203 increases respiration whereas exposure of *Mtb cydC::aph* cells to Q203 show a reduction in OCR as cytochrome bc_1 and CytBD are nonfunctional. In contrast, complementation of *Mtb cydC::aph* with a WT copy of *cydC* restored the OCR to near WT levels (**B** and **C**) when exposed to Q203. Furthermore, exposure of *Mtb* strains to 25 µM GYY4137 (**D**) or 100 µM GYY4137 (**E**) significantly reduced OCR compared to WT *Mtb* and the *Mtb cydC::aph-Comp* clone, demonstrating complementation. Experiments were performed independently at least twice. ΔOCR; the OCR for the media control was subtracted from the OCR for each condition (X-axis). Data shows mean ± SD (n=3-6 per group). Statistical differences (**C**-**E**) were determined using one-way ANOVA with Tukey's multiple comparisons test. *$P < 0.05$, **$P < 0.01$, ***$P < 0.001$, ****$P < 0.0001$

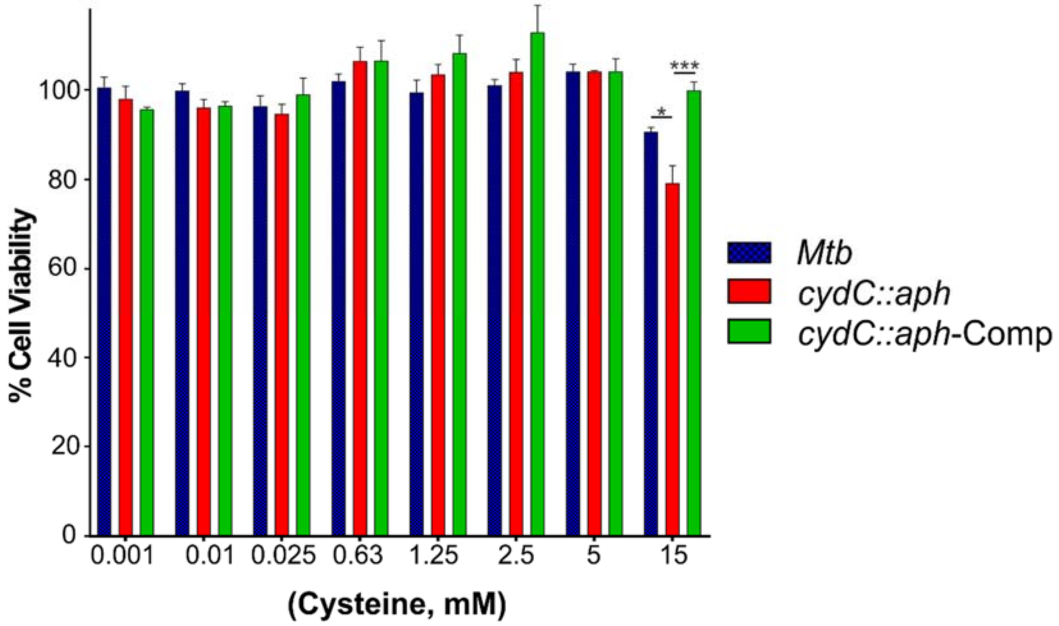

Supplementary Figure 9. Contribution of *Mtb* CydC to resistance against cysteine. *Mtb* cells were treated with various concentrations of cysteine followed by exposure to Alamar blue. Physiological concentrations of cysteine [~128 μM and ~250 μM in human cells and plasma, respectively; (*42, 43*)] do not affect *Mtb* viability. Significant differences were observed only at 15 mM Cys between the *cydC::aph* and *Mtb* groups, and *cydC::aph*-Comp and *cydC::aph* groups. Data are shown as ±SEM (n=3 per group). Data was analyzed using two-way ANOVA with Tukey's multiple comparisons test. *$P < 0.05$, ***$P < 0.001$.

A

Gene ID	Fold change	Description
rv0061c	1.67	Hypothetical protein
rv0079	2.18	Hypothetical protein
rv0080	2.83	Hypothetical protein
rv0081	1.56	HTH-type transcriptional regulator
rv0186	3.31	Beta-glucosidase (BglS)
rv0186A	5.92	Metallothionein (MymT)
rv0187	2.38	O-methyltransferase
rv0211	1.54	Phosphoenolpyruvate carboxykinase (PckA)
rv0440	1.55	Molecular chaperone GroEL2
rv0467	1.51	Isocitrate lyase 1
rv0511	-1.51	Uroporphyrin-III C-methyltransferase (HemD)
rv0512	-1.56	Delta-aminolevulinic acid dehydratase (HemB)
rv0513	-1.66	Transmembrane protein
rv0514	-1.67	Transmembrane protein
rv0542c	-1.74	2-succinylbenzoic acid--CoA ligase (MenE)
rv0814c	1.90	Putative sulfur metabolism (SseC2)
rv0815c	1.63	Thiosulfate sulfurtransferase (CysA)
rv0846c	2.29	Oxidase
rv0847	4.77	Lipoprotein (LpqS)
rv0848	3.24	Cysteine synthase (CysK)
rv0849	2.93	Membrane transporter
rv0967	1.73	Transcriptional repressor (CsoR)
rv0968	1.64	Hypothetical protein
rv0969	1.51	Copper transporter ATPase V (CtpV)
rv0991c	1.73	Hypothetical protein
rv1013	1.84	Polyketide synthase (Pks16)
rv1014c	1.57	Peptidyl-tRNA hydrolase
rv1461	1.51	Hypothetical protein
rv1535	2.01	Hypothetical protein
rv1592c	2.29	Hypothetical protein
rv1711	-1.52	RNA pseudouridine synthase
rv1733c	2.36	Transmembrane protein
rv1734c	1.75	Hypothetical protein
rv1735c	1.76	Membrane protein
rv1736c	1.99	Nitrate reductase-like protein (NarX)
rv1737c	2.66	Nitrate/nitrite transporter (NarK2)
rv1738	2.64	Hypothetical protein
rv1739c	2.98	Sulfate ABC transporter permease
rv1806	1.70	PE family protein (PE20)
rv1812c	1.55	Putatuve dehydrogenase
rv1813c	4.45	Hypothetical protein
rv1869c	-1.62	Putative reductase
rv1870c	-1.51	Hypothetical protein
rv1996	2.12	Universal stress protein
rv1997	1.74	Cation transporter ATPase F (CtpF)
rv2007c	3.37	Ferredoxin A (FdxA)

Gene ID	Fold change	Description
rv2029c	1.63	6-phosphofructokinase (PfkB)
rv2030c	3.89	Hypothetical protein
rv2031c	1.81	Alpha-crystallin
rv2032	3.02	NAD(P)H nitroreductase
rv2056c	1.68	30S ribosomal protein S14
rv2220	1.72	Glutamine synthetase
rv2243	1.59	Malonyl CoA-acyl carrier protein transacylase
rv2244	2.05	Meromycolate extension acyl carrier protein
rv2245	1.66	3-oxoacyl-ACP synthase 1
rv2428	2.51	Alkyl hydroperoxide reductase subunit C
rv2450c	1.81	Resuscitation-promoting factor (RpfE)
rv2451	2.13	Proline/serine-rich protein
rv2552c	-1.55	Shikimate 5-dehydrogenase
rv2553c	-1.51	Membrane protein
rv2623	3.26	Universal stress protein
rv2624c	1.78	Universal stress protein
rv2625c	1.60	Zinc metalloprotease (Rip3)
rv2626c	3.07	Hypoxic response protein
rv2627c	2.86	Hypothetical protein
rv2628	1.54	Hypothetical protein
rv2629	1.60	Hypothetical protein
rv2822c	1.57	CRISPR type III-associated protein (Csm2)
rv2902c	-1.57	Ribonuclease HII
rv2959c	1.58	Rhamnosyl O-methyltransferase
rv2962c	1.58	PGL biosynthesis rhamnosyltransferase
rv2963	2.61	Integral membrane protein
rv3117	1.52	Thiosulfate sulfurtransferase
rv3118	1.60	Hypothetical protein
rv3127	2.31	Hypothetical protein
rv3130c	1.81	Diacyglycerol O-acyltransferase
rv3131	1.65	NAD(P)H nitroreductase
rv3132c	1.76	Sensor histidine kinase (DosS)
rv3133c	1.94	Transcriptional regulator (DosR)
rv3134c	2.49	Universal stress protein
rv3136	1.61	PPE family protein (PPE51)
rv3136A	1.52	Hypothetical protein
rv3159c	-1.56	PPE family protein (PPE53)
rv3614c	1.60	ESX-1 secretion-associated protein (EspD)
rv3615c	1.77	ESX-1 secretion-associated protein (EspC)
rv3633	1.67	Hypothetical protein
rv3846	1.59	Superoxide dismutase A (SodA)
rv3854c	-1.51	Monooxygenase (EthA)
rv3891c	1.55	ESAT-6 like protein (EsxD)
rv3914	1.51	Thioredoxin (TrxC)
rv3918c	-1.56	Chromosome partitioning protein (ParA)
rv3919c	-1.53	rRNA small subunit methyltransferase G

B

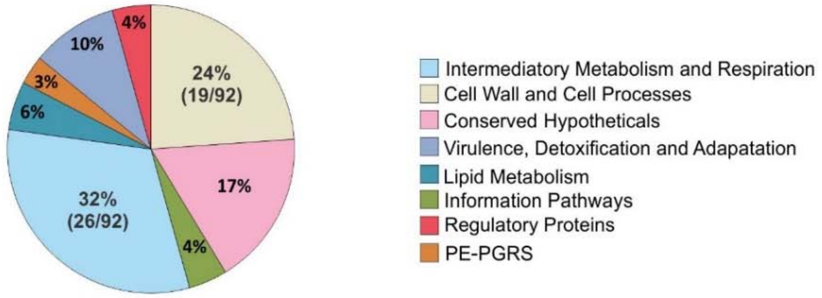

Supplementary Figure 10. Transcriptomic analysis of *Mtb* cells exposed to GYY4137. (**A**) RNAseq based gene expression analysis showing differentially regulated genes in *Mtb* cells following exposure to 25 μM GYY4137 for 1 hr. Each experiment was performed with 3 biological replicates. (**B**) Pie diagram showing differentially regulated genes based on their classification in the Tuberculist database. Numbers in parentheses indicate number of class genes relative to the total number.

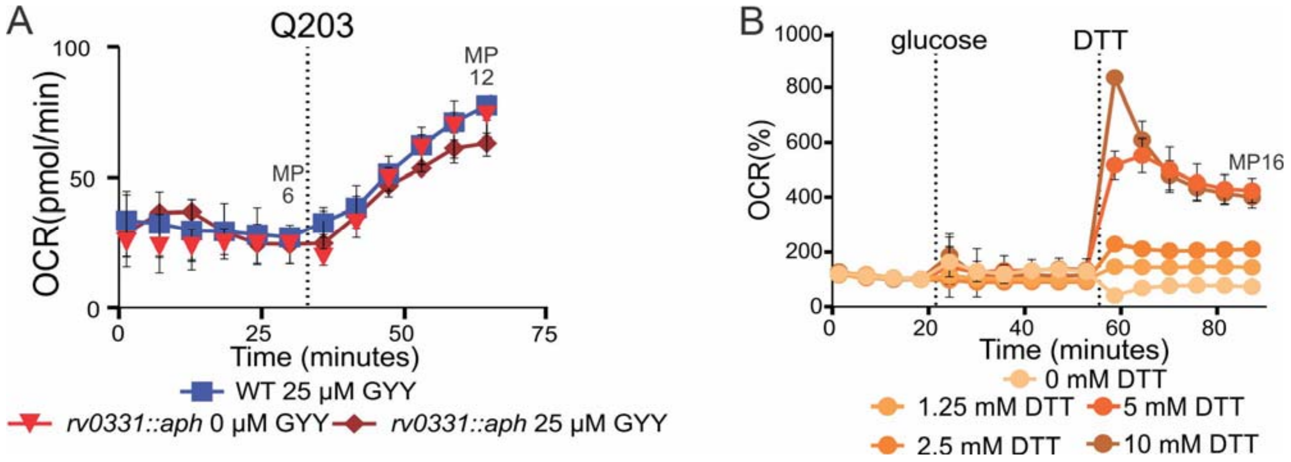

Supplementary Figure 11. Effect of SQR and DTT on *Mtb* OCR. (A) *Mtb sqr* has little effect on OCR in the presence of H_2S. WT *Mtb* CDC1551 and a putative *sqr* mutant strain (*rv0331::aph*; Johns Hopkins library, HG0913, point of insertion at base pair 552 of 1167) was examined in the XF96. GYY4137 was added to the bacilli immediately before the start of the experiment. Six basal respiration measurements were taken before the addition of Q203 (0.3 µM, 100x MIC_{50}), followed by the final six measurements. No significant reduction was observed in the OCR of *rv0331::aph* compared to WT following H_2S addition. **(B)** Percent (%) OCR following addition of glucose and DTT. Four measurements representing basal respiration were taken, followed by glucose addition (final concentration of 0.2%). After glucose addition, six more measurements were taken prior to addition of DTT and the final six measurements were taken following addition of DTT. Following DTT addition, there is an immediate concentration-dependent increase in bacterial respiration. Data are shown as the mean ± SD (n=3-4 per group).

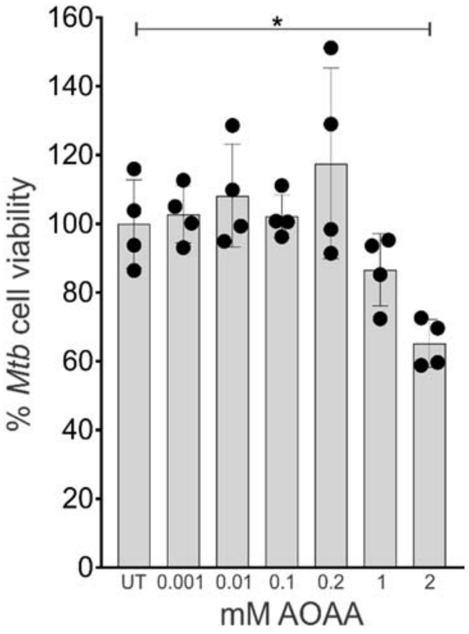

Supplementary Figure 12. Direct effect of AOAA on *Mtb* viability. *Mtb* cells were exposed to various concentrations of AOAA followed by exposure to Alamar blue. Data were normalized to untreated controls, and are shown as mean ±SD (n = 4 per group). Data were analyzed by one-way ANOVA with Tukey's multiple comparisons test.*$P < 0.05$ relative to untreated (UT) control.

Supplementary Table 1. DNA Primers used in this study.

Primer	Sequence (5'-3')
16sRNA-For	GAAGAATGAGCCTGCGAGTC
16sRNA-Rev	GGTCCAGAACACGCCACTAT
hspX-For	CGCACCGAGCAGAAGGAC
hspX-Rev	CCGCCACCGACACAGTAA
fdxA-For	CCTATGTGATCGGTAGTGA
fdxA-Rev	GGGTTGATGTAGAGCATT
rv1813-For	GGTTGCCTATCCCTCCGATTATCC
rv1813-Rev	TCTAGTGCGACTTGCTCTGCTC
rv1620c cydC-For	TATCCATGGTAATGAACCGACCGAGTGCTG
rv1620c cydC-Rev	CTGAAGCTTCGGCGTTACGTGCTGATATCGA
